# Non-Invasive Quantitative Approximation of Intracranial Pressure in Pediatric Idiopathic Intracranial Hypertension Based on Point-of-Care Ultrasound of the Optic Nerve Sheath Diameter

**DOI:** 10.3390/brainsci14010032

**Published:** 2023-12-28

**Authors:** Susanne Regina Kerscher, Julian Zipfel, Andrea Bevot, Nico Sollmann, Karin Haas-Lude, Jonas Tellermann, Martin Ulrich Schuhmann

**Affiliations:** 1 Department of Diagnostic and Interventional Radiology, University Hospital Ulm, 89081 Ulm, Germany; susanne.kerscher@uniklinik-ulm.de; 2 Department of Neurosurgery and Neurotechnology, Division of Pediatric Neurosurgery, University Hospital of Tuebingen, 72076 Tuebingen, Germany; julian.zipfel@med.uni-tuebingen.de (J.Z.); jonas.tellermann@med.uni-tuebingen.de (J.T.); martin.schuhmann@med.uni-tuebingen.de (M.U.S.); 3 Department of Pediatric Neurology and Developmental Medicine, University Children’ s Hospital of Tuebingen, 72076 Tuebingen, Germany; andrea.bevot@med.uni-tuebingen.de (A.B.); karin.haas@med.uni-tuebingen.de (K.H.-L.); 4 Department of Diagnostic and Interventional Neuroradiology, School of Medicine, Klinikum rechts der Isar, Technical University of Munich, 81675 Munich, Germany; 5 TUM-Neuroimaging Center, Klinikum rechts der Isar, Technical University of Munich, 81675 Munich, Germany

**Keywords:** optic nerve sheath diameter, ONSD, ultrasound, idiopathic intracranial hypertension, ICP, mathematical approach, non-invasive

## Abstract

Background: To investigate whether ultrasound-based optic nerve sheath diameter (US-ONSD) is a reliable measure to follow up children with idiopathic intracranial hypertension (IIH). In addition, to analyze the inter- and intra-individual relationships between US-ONSD and intracranial pressure (ICP), and to investigate whether an individualized mathematical regression equation obtained from two paired US-ONSD/ICP values can be used to approximate ICP from US-ONSD values. Methods: 159 US examinations and 53 invasive ICP measures via lumbar puncture (LP) were performed in 28 children with IIH. US-ONSD was measured using a 12 Mhz linear transducer and compared to ICP values. In 15 children, a minimum of 2 paired US-ONSD/ICP determinations were performed, and repeated-measures correlation (rmcorr) and intra-individual correlations were analyzed. Results: The cohort correlation between US-ONSD and ICP was moderate (r = 0.504, *p* < 0.01). Rmcorr (r = 0.91, *p* < 0.01) and intra-individual correlations (r = 0.956–1) of US-ONSD and ICP were excellent. A mathematical regression equation can be calculated from two paired US-ONSD/ICP values and applied to the individual patient to approximate ICP from US-ONSD. Conclusions: Related to excellent intra-individual correlations between US-ONSD and ICP, an individualized regression formula, created from two pairs of US-ONSD/ICP values, may be used to directly approximate ICP based on US-ONSD values. Hence, US-ONSD may become a non-invasive and reliable measure to control treatment efficacy in pediatric IIH.

## 1. Introduction

Idiopathic intracranial hypertension (IIH) is a rare neuropediatric disorder affecting approximately 0.71 in 100,000 children per year [1,2]. It is characterized by elevated intracranial pressure (ICP) with normal cerebrospinal fluid (CSF) composition and magnetic resonance imaging (MRI)-based exclusion of hydrocephalus or space-occupying lesions [3,4]. Patients typically present with headaches, eye pain, nausea, vomiting, dizziness, or sometimes visual disturbances (e.g., double vision, blurred vision). Initial diagnosis usually requires invasive measurements of the ICP via lumbar puncture (LP) [4,5].

Therapeutic options include medications (usually with acetazolamide, steroids, or furosemide), repeated LP with CSF depletion, ventriculo- or lumbo-peritoneal shunt systems, and venous sinus stenting in cases of severe and therapy-refractory IIH [6,7,8]. In patients with obesity, weight reduction is usually the first and most important therapeutic option, whereas bariatric surgery is reserved for adults in cases of treatment resistance [6]. Close monitoring of treatment efficacy is extremely important, as untreated and/or recurrent IIH can lead to severe visual impairment and even blindness [9]. Likewise, untreated IIH is a serious health burden for patients, can lead to cognitive impairment, and, in extreme cases, can be fatal [5,8]. Monitoring of the therapy is often based on repeated measurements of ICP by LP or fundoscopy of the ocular fundus. However, the latter is associated with limited sensitivity in children to detect increased ICP in IIH, as papilledema may be absent in children with elevated ICP in up to 50% of cases [10,11,12,13]. Invasive LP is not only painful but also carries the risk of infection and requires sedation, at least in children, which in turn is associated with increased overall effort and other risks such as the suspected long-term neurocognitive effects of multiple sedations on the developing brain [14].

Transorbital point-of-care ultrasound-based optic nerve sheath diameter (US-ONSD) is a non-invasive and reliable measure for estimating ICP in both children and adults with multiple applications [15,16,17]. A good correlation between ONSD and invasively measured ICP values has been described, suggesting a general linear relationship between these two parameters [18,19]. However, there is evidence that individual factors, such as the underlying diseases, degree and duration of ICP elevation, individual intracranial compliance, and individual hysteresis can strongly influence the relationship between ONSD and ICP [20].

Against this background, the aim of this study was to analyze the overall inter-individual correlation between US-ONSD and ICP in pediatric IIH. In addition, the common intra-individual associations for paired US-ONSD/ICP measurements were investigated by repeated-measures correlation (rmcorr). Furthermore, an analysis of the intra-individual correlation between US-ONSD and ICP in individual children with IIH was performed. Finally, this study also aimed to provide a general mathematical regression formula for the approximate calculation of ICP from US-ONSD values.

## 2. Materials and Methods

### 2.1. Study Design and Sample Size Calculation

This single-center, prospective, observational study collected data on children diagnosed with IIH. Pediatric patients (2018–2022) were included if they had undergone both a transorbital US-ONSD determination and an invasive ICP measurement. Other inclusion criteria included a definite diagnosis of IIH according to the revised Friedman criteria [4]: (1) clinical symptoms of increased ICP (e.g., headache, eye pain, nausea, vomiting, dizziness, visual disturbances such as double or blurred vision), (2) LP opening pressure ≥18.5 mmHg/25 cmH_2_O (≥21 mmHg/28 cmH_2_O if the child was sedated and/or obese) without cytological CSF abnormalities, (3) absence of hydrocephalus, space-occupying lesions, or venous sinus thrombosis on MRI, and (4) no evidence of secondary causes of IIH. Patients who had previously undergone ONS fenestration or were additionally diagnosed with diseases potentially associated with ON swelling, such as optic neuritis or optic glioma, were excluded from this study. The patients were examined during in-house stays or on an outpatient basis.

The sample size estimate for the correlation of US-ONSD and ICP was calculated at 24 patients to achieve 90% power with an alpha of 5 (*p* < 0.05). With an estimated loss of 10% (e.g., due to the study exclusion criteria and data loss), the study aimed to recruit a minimum of 27 patients.

### 2.2. Study Population

A total of 28 children aged 1 to 17 years (mean age 9.4 ± 4.4 years) were included. Among those, 19 (67.9%) children were male, and 9 (32.1%) children were female.

All patients presented with clinical symptoms of elevated ICP and underwent US-based measurement of the ONSD and an invasive ICP measurement by LP at baseline. All patients were diagnosed with IIH according to the abovementioned criteria [4].

After diagnosis, patients were assigned to therapy (medical therapy with acetazolamide ± furosemide), and 15/28 children underwent a series of 2–7 repeated, paired US-ONSD and invasive ICP measurements by LP ± CSF depletion because of clinical symptoms and findings or initially very high ICP. Some patients responded to therapy with a gradual decrease in ICP, while others relapsed or required a dose increase. The main question of this study, the intra-individual correlation between US-ONSD and ICP at any point in time, included all individual patient and treatment factors (such as type and duration of therapy, relapse etc.), given that all these factors can significantly influence the respective ICP. A total of 159 US-ONSD examinations and 53 invasive ICP measurements by LP were performed.

### 2.3. US Investigation

One examiner (S.R.K.) with 5 years of experience in transorbital US performed all US examinations. All baseline examinations were performed before LP; the time interval between US and LP was less than 10 min. If sedation was required for LP, this was administered before the US examination so that both US and LP were performed under the same conditions. The US examination after CSF depletion was performed directly after LP and CSF depletion either with or without sedation, depending on the conditions under which the LP was performed. The investigator was blinded to the clinical information/diagnosis.

**Figure 1:**
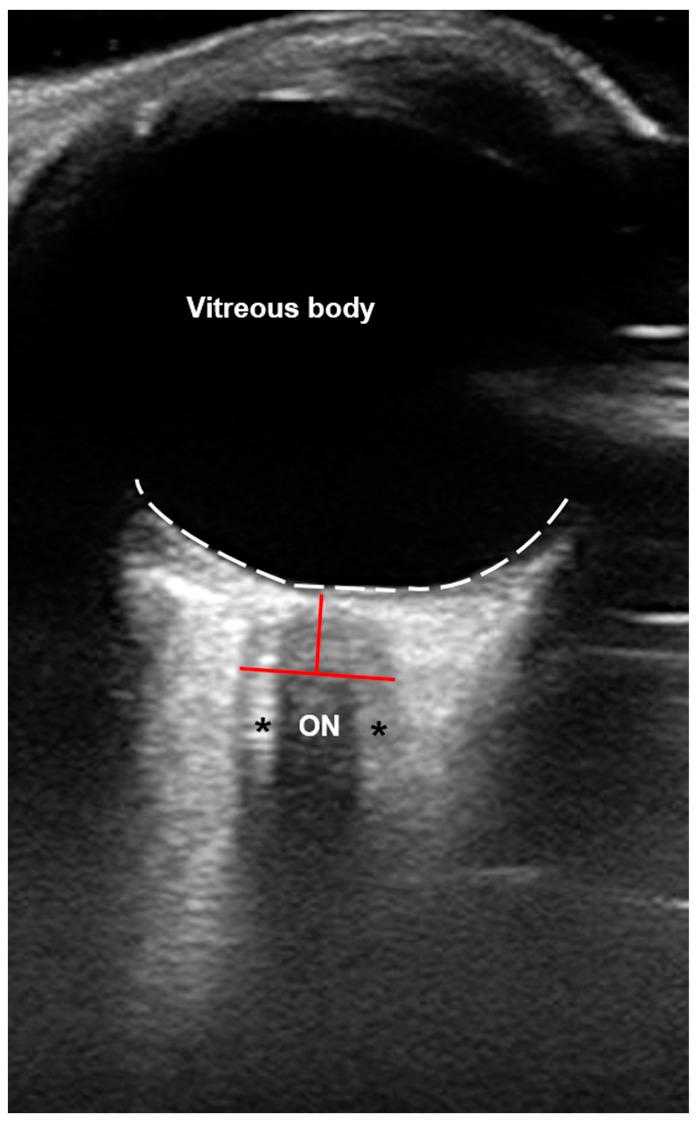
**Axial B-scan ultrasound (US) of the orbit.** The optic nerve (ON) appears hypoechogenic and is surrounded by the ON sheath (ONS; black asterisks) that encases the optic subarachnoid space (SAS), which is filled with cerebrospinal fluid (CSF) and subdivided by multiple trabeculae [22], presenting with a hyperechogenic appearance. The white dotted line marks the retina. Red lines mark the exact region to measure the ONS diameter (ONSD) at about 3 mm posterior to the ON head.

The US examination was performed on patients in supine position, with the head straight and not elevated. Specifically, US-ONSD was examined in B-mode with a 12 MHz linear transducer (Epiq 5G US system; Philips Healthcare, Best, The Netherlands), and ONSD was measured 3 mm posterior to and at a 90° angle to the ON (Figure 1). Three measurements were taken in the axial plane, and the mean ONSD of each side and the resulting mean binocular US-ONSD were calculated as previously described [21]. Images were stored in joint photographic experts group (JPEG) and digital imaging and communications in medicine (DICOM) formats.

### 2.4. Invasive ICP Measurement by LP

The ICP measurement was obtained through a LP, which was performed by neuro-pediatricians blinded to the US results. Patients were placed in a lateral position, with knees and hips flexed and the head as close to the knees as possible. If sedation was necessary, drugs suspected of influencing the ICP (e.g., ketamine) were not used. In cases where sedation was not required, local anesthesia was given prior to LP using a cream/plaster containing lidocaine and/or lidocaine infiltration. For the ICP measurement after successful puncture of the subarachnoid space (SAS) between the spinous processes of the lumbar vertebrae 3–5, the patient’s position was relaxed, the legs and trunk were straightened, and a neutral head position was assumed in order to minimize the influence of the position on the CSF pressure. The guide needle was carefully removed, ensuring that no CSF escaped before the measurement. A riser tube was then connected to the inserted cannula under sterile conditions, and the initial ICP was measured. The riser tube was then removed again, and, depending on the opening pressure and the age of the patient, up to 30 mL of CSF was drained. Finally, the riser tube was reconnected to the cannula in a sterile manner, and the ICP was measured a second time. For better comparability with other studies in which ICP was measured (e.g., by an intraparenchymal probe or external ventricular drainage) and expressed in mmHg, ICP was converted from cmH_2_O to mmHg, based on the fact that the pressure unit of 1 cmH_2_O corresponds to 0.735539 mmHg (https://physics.nist.gov/cuu/pdf/sp811.pdf, last accessed on 12 December 2023).

### 2.5. Statistical Analysis

Statistical analyses were performed using SPSS software (version 29; IBM Corp., Armonk, NY, USA). Data were tested for normality of distribution using the Shapiro–Wilk test. Parametric data were expressed as the mean and standard deviation (sd). Based on the data distribution, correlation analysis was performed according to Pearson or Spearman correlation analyses. Rmcorr was performed to determine associations for paired US-ONSD/ICP measures (assessed on two or more occasions for multiple individuals). Power analysis was used to calculate sample sizes. Mathematical formulas were derived based on the general linear regression equation y = mx + t (t = intercept with the *y*-axis, m = estimated coefficient for the linear term). Statistical significance was set at *p* < 0.05. Correlation coefficients were considered weak when the correlation coefficient r was less than 0.4, moderate when r was between 0.4 and 0.59, strong when r was greater than 0.6, and excellent when r was greater than 0.8.

## 3. Results

### 3.1. Inter-Individual Correlation of US-ONSD and ICP

**Figure 2:**
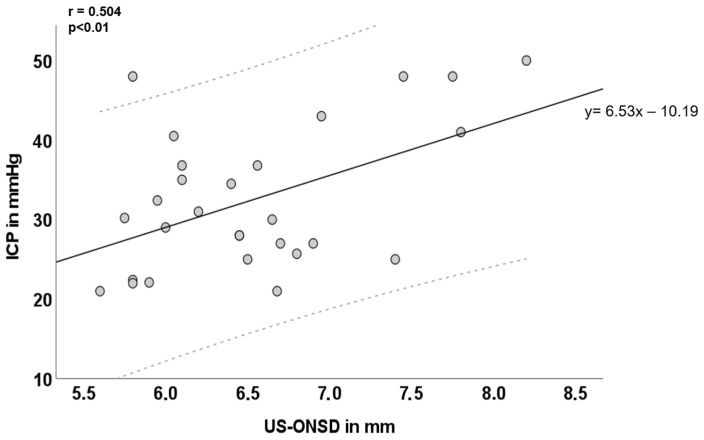
**Inter-individual correlation of ultrasound-based optic nerve sheath diameter (US-ONSD) and intracranial pressure (ICP) over the entire cohort.** Light grey dashed lines mark the 95% confidence interval (CI). Y = 6.53 x – 10.19 is the corresponding overall regression equation; *n* = 28.

In 28 children presenting with signs or symptoms of elevated ICP, the mean baseline US-ONSD was 6.5 ± 0.7 mm and ranged from 5.6–8.2 mm. In 2/56 eyes of 2 different children, (partial) optic atrophy was diagnosed. US-ONSD did not differ significantly between optic atrophic and non-atrophic eyes: patient 1—atrophic eye US-ONSD: 6.4 mm, non-atrophic eye US-ONSD: 6.3 mm, patient 2—atrophic eye US-ONSD: 6.0 mm, non-atrophic eye US-ONSD: 6.1 mm. Mean ICP at initial LP was 32.4 ± 9.0 mmHg and ranged from 21–50 mmHg. Both US-ONSD and ICP values were normally distributed and moderately correlated (r = 0.504, *p* < 0.01; Figure 2). The general regression equation was calculated (y = 6.53 x − 10.19) and the regression line including a 95% confidence interval (CI) was inserted. Furthermore, 27/28 (96.4%) of the values were within the CI. Since y was equal to ICP and x was equal to US-ONSD, it would, in principle, be possible to approximate ICP from individual US-ONSD values based on the regression equation. However, this does not take into account intra-individual factors (e.g., age, duration and extent of ICP elevation, individual intracranial compliance, and dural elasticity of the ONS) that influence the relationship between ICP and ONS expansion.

### 3.2. Rmcorr and Intra-Individual Correlation of US-ONSD and ICP

**Figure 3:**
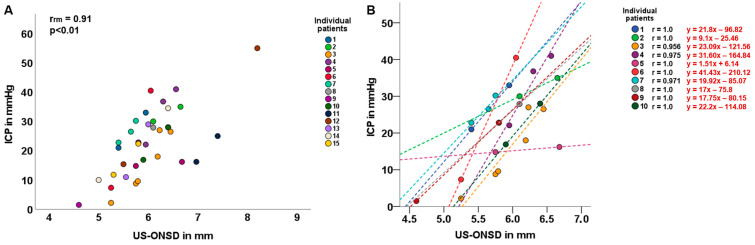
**Common intra-individual association and intra-individual correlation of ultrasound-based optic nerve sheath diameter (US-ONSD) and intracranial pressure (ICP).** (**A**) Repeated-measures correlation (rmcorr; r_rm_) of US-ONSD/ICP in 15 children with idiopathic intracranial hypertension (IIH). (**B**) Intra-individual correlation of US-ONSD and ICP in 10 children with IIH. Differently colored dashed lines show individual regression lines for different patients.

A total of 2–7 repeated measurements of US-ONSD and concurrent invasive ICP assessments were performed in 15/28 children. The common intra-individual association for paired US-ONSD/ICP measures for multiple individuals was determined by rmcorr (r_rm_ = 0.91, *p* < 0.01; Figure 3A).

In 10/15 children, an analysis of the intra-individual correlation between US-ONSD and ICP was performed, yielding very high correlation coefficients ranging from 0.956 to 1.0 (Figure 3B). For each patient, the individual regression equation was calculated, and the regression line was plotted.

### 3.3. Mathematical Regression Equation to Describe the Intra-Individual Relationship between ICP and ONSD

The above results suggest a general relationship between ICP and ONSD in patients with pediatric IIH, although the intra-individual characteristics differ significantly from those of the cohort. Therefore, it may be only possible, in individual patients, to approximate ICP from US-ONSD values using a regression equation. Such an individual regression equation can be established once two paired US-ONSD/ICP measurements have been obtained from a single patient (e.g., in the context of the initial diagnosis of IIH and the required LP using baseline and post-CSF depletion measurements of US-ONSD and ICP).

**Figure 4:**
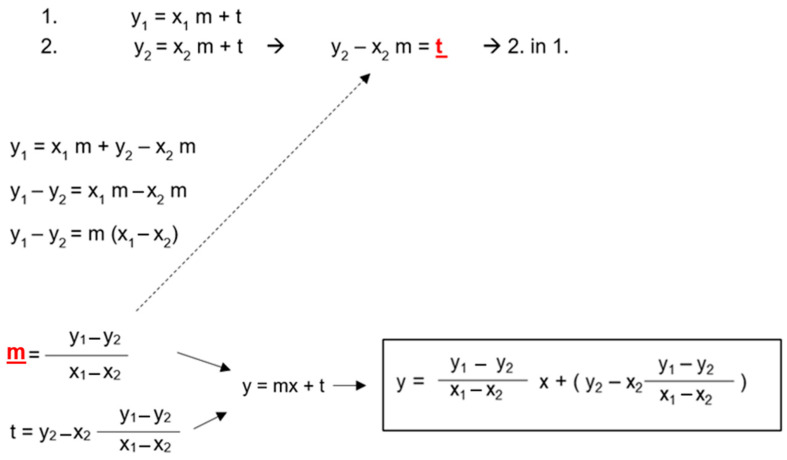
**Derivation of a general mathematical formula for the calculation of intracranial pressure (ICP) from two pairs of ultrasound-based optic nerve sheath diameter (US-ONSD)/ICP values obtained during the initial diagnosis of pediatric idiopathic intracranial hypertension (IIH).** x_1_ = US-ONSD_1_ (baseline ONSD before LP), x_2_ = US-ONSD_2_ (ONSD after CSF depletion). y_1_ = ICP_1_ (baseline ICP at LP), y_2_ = ICP_2_ (ICP after CSF depletion). t and m (red fonts) can be calculated using the rearranged equations by substituting the two pairs of values. To establish the individual formula for the patient, t and m are finally inserted into the general linear equation y = mx + t.

The regression equation for the linear model takes the following form: y = mx + t, where t is the constant or intercept with the *y*-axis and m is the estimated coefficient for the linear term (i.e., the slope of the line). Therefore, only t and m are needed to obtain an individual regression equation for each patient (Figure 4). So, if

y_1_ = ICP_1_ (baseline ICP at LP) x_1_ = US-ONSD_1_ (baseline ONSD before LP) y_2_ = ICP_2_ (ICP after CSF depletion) x_2_ = US-ONSD_2_ (ONSD after CSF depletion)

## 4. Discussion

This study investigated US-ONSD and invasive ICP values in children with IIH. The study also performed the common intra-individual association for paired US-ONSD/ICP measurements for multiple individuals and an analysis of the intra-individual correlation of US-ONSD and ICP in pediatric IIH. From the collected results, the present work derived a general mathematical formula applicable to individual patients in everyday clinical practice to non-invasively approximate the corresponding ICP value from US-ONSD values.

### 4.1. Inter- and Intra-Individual Relationships between ONSD and ICP

Transorbital US of the ONSD is a non-invasive, easy-to-learn method to estimate ICP with high intra- and inter-rater reliability [15,23]. The US-ONSD-based assessment of ICP has many different applications in adults and children, e.g., in traumatic brain injury, hydrocephalus, tumors, and IIH [24,25,26,27]. The wide availability of US machines, ease of setup, and cost effectiveness make US-ONSD a valuable point-of-care technology, not only in countries with sophisticated medical systems but especially in middle- and low-income countries [28]. A number of studies have investigated the relationship between ONSD and ICP in both children and adults [16,17,18]. In studies of pediatric patients, there is some controversy regarding the correlation between ONSD and ICP, with some describing an intermediate to high correlation while others found a weak or no clear correlation [15,16,29,30]. In general, there is an age dependence in children, with a good correlation (with r around 0.63 [16] to 0.66 [15]) in children over 1 year of age and a poor correlation in very young children with patent fontanels [16,18]. There also appears to be a poor correlation in children in the intensive care setting [30]. Regarding pediatric IIH, there are a few studies with controversial results that have performed correlation analyses between ONSD and ICP; for example, Irazuzta et al. showed a very high correlation (r > 0.9) between ONSD and ICP in 13 children in a prospective study of pediatric IIH [31]. Another recent prospective study of 8 children diagnosed with IIH showed a weak and non-significant correlation (r = 0.298, *p* > 0.05) between ONSD and invasively measured ICP [32]. Aslan et al. published a study of 7 children with IIH and good correlations between ONSD and ICP, with a correlation coefficient r ranging from 0.649 to 0.882 [27]. Our study found an overall moderate inter-individual correlation between ONSD and ICP in pediatric IIH in 28 children, with r = 0.504.

Given the anatomical and physiological conditions, it seems obvious that there is a general linear relationship between ONSD and ICP. The intracranial SAS (iSAS) is directly connected to the optic SAS (oSAS), so that changes in ICP are immediately transmitted from the iSAS to the oSAS [22]. Thus, an increase in pressure in the intracranial compartment is followed by a rapid dilatation of the dural and elastic ONS [33]. However, it has been shown that a large increase in ICP is not necessarily associated with a large expansion of the ONSD and vice versa [20]. In fact, it has been shown that in childhood IIH, compared to other neuropediatric findings, such as hydrocephalus, arachnoid cysts, or brain tumors, changes in ICP are highly correlated with changes in ONSD [20]. This may be due to the fact that the pathophysiology of IIH is relatively uniform compared to the pathophysiology of hydrocephalus or brain tumors. Nevertheless, many individual factors, such as the type of disease, duration, degree of ICP elevation, type of treatment, and individual intracranial compliance, appear to influence the degree of ONSD enlargement in relation to the degree of ICP elevation [20]. Therefore, it is not appropriate to describe the relationship between ICP and ONSD enlargement in a general linear regression equation without considering individual circumstances. Based on these considerations, we first performed an analysis of the common intra-individual association for paired repeated measures of US-ONSD/ICP. This calculation showed a high rmcorr with r_rm_ = 0.91. The rmcorr plot (Figure 3A) shows that there is a strong intra-individual ONSD-ICP association in children with IIH, taking into account the independence of observations and the different patterns between and within participants. Rmcorr estimates the joint regression slope, i.e., the joint association between individuals [34].

In a second step, we performed intra-individual correlation and regression analyses in 10 children for whom repeated measures of paired US-ONSD/ICP values were available. An individual regression equation was developed for each child, and the 10 regression lines were fitted to the graph (Figure 3B). The 10 correlation analyses between US-ONSD and ICP yielded excellent values ranging from r = 0.956 to r = 1.0. The high variability of the regression lines confirms the above considerations of individual factors influencing the relationship between ONSD and ICP.

To illustrate the importance of an individual equation per patient, consider patient 3 (orange line and dots, Figure 3: The corresponding individual regression equation is: y = 23.09 x − 121.56, based on ICP values measured in mmHg). Suppose this patient asymptomatically presents for clinical follow-up exams and an US-ONSD of 5.6 mm is measured. Substituting this US-ONSD into the above regression equation would yield y (ICP) = 23.09 * 5.6 − 121.56 = 7.74 mmHg (as compared to the graph in Figure 3B). Applying the general regression equation for the entire cohort (Figure 2, y = 6.53 x − 10.19) would yield an estimated ICP of 26.38 mmHg. This discrepancy demonstrates that individual patient factors and circumstances must be taken into account and that a general “IIH formula” may be inadequate for ICP estimation. Hence, these results suggest that the US-ONSD in pediatric IIH could be an excellent tool for outcome assessment, even providing a quantitative result once an individual regression equation has been established for the patient.

### 4.2. Use of a General Mathematical Formula to Approximate Individual ICP Values from US-ONSD

Effective and reliable monitoring of therapy in IIH remains a challenge. Non-invasive fundoscopy of the ocular fundus by a qualified examiner is considered the reference standard for detecting or excluding elevated ICP and is also used to monitor IIH therapy in children [2]. However, fundoscopy is known to have limited sensitivity for detecting elevated ICP in children [35,36]. In particular, the absence of papilledema does not exclude elevated ICP [12]. In addition, the resolution of papilledema after ICP reduction or normalization takes weeks to months [37,38], and therefore the persistence of papilledema does not necessarily indicate that the therapy is ineffective. Another regularly performed method to monitor treatment efficacy is repeated LP. In addition to being painful, LP is associated with a risk of infection and requires sedation in most children [14]. Therefore, especially in IIH, which is associated with long treatment periods [39], a non-invasive, easy-to-perform, and reliable method of follow-up evaluation is needed.

Robba et al. validated a general regression formula for direct estimation of ICP from US-ONSD values in a cohort of adult brain-injured patients [40]. When the group applied this formula to children with traumatic brain injury, it became apparent that the use of this general formula in the pediatric cohort was not recommended, primarily because of the high degree of variability in ONSD and ICP. In this cohort of Robba et al., there was a clear individual relationship between ONSD and ICP as well. The formula also led to an underestimation of the true ICP [41].

On the other hand, the creation and application of an individual regression equation in clinical practice is a readily conceivable and feasible option, especially in IIH. To establish such an equation for an individual patient, at least two pairs of US-ONSD/ICP values are required. Since patients with IIH usually receive at least one LP with measurement of the opening pressure as part of the diagnostic workup, two pairs of values are obtained before and after CSF drainage. Of course, the ICP must be consistently expressed in mmHg or cmH_2_O, as must the ONSD in mm or cm. The easiest way to arrive at such an equation is to first calculate the slope (m) of the equation using the two pairs of US-ONSD/ICP values and then, in a second step, to calculate t, the constant or the intercept with the *y*-axis. Finally, m and t are substituted into the general equation (y (ICP) = m * x(ONSD) + t) to obtain the individual regression equation for the patient. This equation can then be used for future follow-up examinations with US-ONSD. According to the approximated ICP values, the diagnostic steps and therapy can then be adjusted during the course of treatment, and unnecessary invasive LP can be avoided. For the sake of simplicity, we recommend the automatic calculation of the parameters in clinical routine with the help of a standardized spreadsheet prepared accordingly beforehand.

### 4.3. Limitations

The main limitation is the small sample size of this observational study, despite its prospective nature. Larger and ideally multicenter cohorts should be studied to be able to recommend the general use of an individual regression equation for direct estimation of ICP from ONSD values in childhood IIH. It should also be noted that in this study we focused exclusively on how a patient’s individual ICP value correlated with the corresponding US-ONSD at each individual time point, as we wanted to derive the formula for calculating ICP from ONSD values from these results. In the clinical setting, ICP values and their various clinical manifestations (which are often non-specific in children [42,43]) are the main focus of the diagnostic work-up and management of IIH patients, as they determine further diagnostic and therapeutic steps. However, it would be interesting for future clinical studies to investigate how, for example, the type of therapy or the development of papilledema affects the course of US-ONSD. Another limitation is that the overall context of the relationship between ONSD and ICP is not yet fully clarified or understood to the last detail. Finally, our ICP measurements were all performed with LP. The quality of the ICP measurement by LP depends on several parameters, such as patient positioning, sedation, and the experience of the operator. Therefore, studies including different methods of ICP measurement could be helpful to enforce the validity of an individualized formula.

## 5. Conclusions

In pediatric IIH, the US-ONSD showed a moderate inter-individual cohort correlation to ICP, but the intra-individual correlation of US-ONSD and ICP was high, with individual slopes of the regression lines indicating a strong influence of individual factors on the correlation of ONSD and ICP. An individualized mathematical regression equation derived from two pairs of US-ONSD/ICP values (e.g., obtained during the first diagnostic LP) may thus be used in daily clinical practice to approximate the corresponding ICP value non-invasively and directly from US-ONSD values during follow-up examinations. Such a non-invasive quantitative assessment tool would have great potential to supplement or even replace invasive follow-up methods in pediatric IIH, pending confirmation of our results by future large-scale and ideally multicenter studies.

## Data Availability

The data are not publicly available due to restrictions e.g., their containing information that could compromise the privacy of research participants. The datasets generated during and/or analyzed during the current study are available from the corresponding author upon reasonable request.
